# Forecasting COVID-19 Cases Using Alpha-Sutte Indicator: A Comparison with Autoregressive Integrated Moving Average (ARIMA) Method

**DOI:** 10.1155/2020/8850199

**Published:** 2020-12-03

**Authors:** A. M. C. H. Attanayake, S. S. N. Perera

**Affiliations:** ^1^Department of Statistics & Computer Science, Faculty of Science, University of Kelaniya, Sri Lanka; ^2^Research & Development Centre for Mathematical Modelling, Department of Mathematics, Faculty of Science, University of Colombo, Sri Lanka

## Abstract

COVID-19 is a pandemic which has spread to more than 200 countries. Its high transmission rate makes it difficult to control. To date, no specific treatment has been found as a cure for the disease. Therefore, prediction of COVID-19 cases provides a useful insight to mitigate the disease. This study aims to model and predict COVID-19 cases. Eight countries: Italy, New Zealand, the USA, Brazil, India, Pakistan, Spain, and South Africa which are in different phases of COVID-19 distribution as well as in different socioeconomic and geographical characteristics were selected as test cases. The Alpha-Sutte Indicator approach was utilized as the modelling strategy. The capability of the approach in modelling COVID-19 cases over the ARIMA method was tested in the study. Data consist of accumulated COVID-19 cases present in the selected countries from the first day of the presence of cases to September 26, 2020. Ten percent of the data were used to validate the modelling approach. The analysis disclosed that the Alpha-Sutte modelling approach is appropriate in modelling cumulative COVID-19 cases over ARIMA by reporting 0.11%, 0.33%, 0.08%, 0.72%, 0.12%, 0.03%, 1.28%, and 0.08% of the mean absolute percentage error (MAPE) for the USA, Brazil, Italy, India, New Zealand, Pakistan, Spain, and South Africa, respectively. Differences between forecasted and real cases of COVID-19 in the validation set were tested using the paired *t*-test. The differences were not statistically significant, revealing the effectiveness of the modelling approach. Thus, predictions were generated using the Alpha-Sutte approach for each country. Therefore, the Alpha-Sutte method can be recommended for short-term forecasting of cumulative COVID-19 incidences. The authorities in the health care sector and other administrators may use the predictions to control and manage the COVID-19 cases.

## 1. Introduction

COVID-19 or novel coronavirus disease is one of the fastest spreading diseases around the world. It is an infectious disease that occurred by a newly identified strain of coronavirus. The World Health Organization (WHO) stated this disease as a pandemic on February 11, 2020. The disease was first identified in Wuhan City of China during December 2019. Currently, it is a threat facing more than 200 countries in the world. 967,164 confirmed deaths and 31,425,029 confirmed cases have been reported around the world on September 23, 2020 [1]. Nearly over two hundred thousand new cases and over five thousand new deaths are reported daily in the world. These figures illustrate how far the disease spread among humans and the severity of the disease.

The USA, India, and Brazil are the top three countries in the world which recorded the highest number of total COVID-19 cases. The total number of cases reported in the USA was 7,139,553 and in India was 5,730,184, whereas in Brazil was 4,627,780 on September 23, 2020 [2]. The highest number of daily new COVID-19 cases was reported in India on September 23, 2020, followed by the USA and then Brazil. These three countries have obtained the attention of the community because of their active engagement with the COVID-19. On March 19, 2020, Italy was the country where the highest number of deaths were depicted in the world. Currently, Italy has recorded continuous cases during their second wave of the epidemic. 35,781 deaths have been reported with only 23 new deaths in Italy on September 24, 2020. The beginning of COVID-19 was recorded in New Zealand on February 28, 2020 [3]. After six months from February, the total number of COVID-19 cases recorded in New Zealand has been around 1,800 cases, showing that the country is implementing its control mechanisms effectively. Only 25 deaths have been reported in the country. Therefore, New Zealand is at the ending phase of the epidemic. South Africa has reported continuous progression with the number of COVID-19 cases. It is the country which has recorded the highest number of total cases in the African region. 663,282 cases have been reported with 16,118 deaths in South Africa as of September 22, 2020. 663,556 cases tested positive for coronavirus in Spain on September 23, 2020. Spain is in the second wave of the epidemic and has reported 32,159 deaths so far. The county holding the third position in terms of the overall COVID-19 cases is the European region. 306,886 cases are reported in Pakistan as of September 22, 2020. Around 6,400 deaths are reported within the country. It is within the list of the top ten countries in Asia which are affected with COVID-19. Pakistan appears to be reaching to the ending phase of the COVID-19. Clearly, the mentioned eight countries are in one of the growing, controlling, and ending phases of the COVID-19, we decided to select these countries for our analysis.

Further, the selected eight countries represent different socioeconomic and geographical backgrounds (Table 1). Other than Antarctica, at least one country from each of the other continents was included in the study. Human development index can be considered as a measure of human development in a country, and it is based on education, health, and life expectancy. If the score is above 0.8, then the country can be considered as a developed country. The selected sample consists of developed and developing countries. The economic impact due to COVID-19 differs across various countries in the sample. The governments of some countries are implementing supportive policies to manage COVID-19 while others totally ignore such policies. Therefore, the selected sample of countries compromises with diverse socioeconomic and geographical backgrounds as well as in different growth stages of the epidemic.

Statistical techniques can be applied to model and predict the transmission of COVID-19. The predictions generated should be directed to implement controlling strategies as well as to take appropriate decisions to alleviate the impact of COVID-19. The applications of time-series approaches in modelling and predicting COVID-19 can be found in the literature [4–7]. Among several univariate time-series approaches, Autoregressive Integrated Moving Average (ARIMA) and Exponential Smoothing techniques are widely applied for modelling purposes. These techniques are appropriate in short-term forecasting, and more than 50 observations are required in the modelling process to capture the patterns existing in the data series accurately. This study aims to model and predict COVID-19 cases. Particularly, COVID-19 cases evolved in the eight countries: Italy, New Zealand, USA, Brazil, Pakistan, Spain, South Africa, and India, were selected as test cases. The Alpha-Sutte Indicator approach was utilized as the modelling strategy. It is one of the powerful, effective, and newly invented strategies [8, 9] for modelling time series data. The capability of the method in modelling COVID-19 cases over the ARIMA method was tested in the current study.

## 2. Materials and Methods

### 2.1. Data and Methodology

Daily cumulative COVID-19 cases evolved in Italy, New Zealand, USA, Brazil, Pakistan, Spain, South Africa, and India were acquired [10] from the first day of the presence of cases to September 26, 2020. Data recording of the countries was started on different dates and is summarized in Table 2. The data set is divided into two portions: as one part for the training of the models and the other for the validation. The details of the training data are given in Table 2.

The data are modelled using ARIMA and Alpha-Sutte approaches. The last 10% of the data were used to validate the approaches. The differences between the actual and predicted COVID-19 cases in the validation set of each country were tested using the paired *t*-test. The analysis of the study was done using the R software package [11]. The models were formed by utilizing functions in the stats, forecast, graphics, MLmetrics, and xlsx packages of the R software.

### 2.2. Techniques and Definitions

#### 2.2.1. Alpha-Sutte Indicator

The Alpha-Sutte method was developed to forecast the data on finance, insurance, and time-series data and originated from the Sutte Indicator [8]. One of the main advantages of this modelling strategy is it requires only 4 observations to model and can predict the fifth observation. Therefore, this modelling approach is particularly suitable for short-term forecasting of data series. Further, no assumptions are existing to validate the model. The formula of the Alpha-Sutte method is shown below [9]:

(1) $$\begin{array}{c} a_{t}=\frac{\alpha \left(∆x/\left(\left(\alpha +\delta \right)/2\right)\right)+\beta \left(∆y/\left(\left(\alpha +\beta \right)/2\right)\right)+\gamma \left(∆z/\left(\left(\beta +\gamma \right)/2\right)\right)}{3}, \end{array}$$

where *δ* = *a*_*t*−4_, *α* = *a*_*t*−3_, *β* = *a*_*t*−2_, *γ* = *a*_*t*−1_

(2) $$\begin{array}{c} ∆x=\alpha -\delta ,∆y=\beta -\alpha ,∆z=\gamma -\beta , \end{array}$$

*a*_*t*_ is the observation at time *t*.

#### 2.2.2. Autoregressive Integrated Moving Average (ARIMA) Method

ARIMA is a univariate time series modelling approach which is used to capture the patterns in the data in order to forecast the future values of the series. ARIMA model usually denoted as ARIMA (*p*, *d*, *q*), where *p* stands for autoregressive (AR) parameter, *q* for moving average (MA) parameter, and *d* for the order of differencing. There are basically three stages in the ARIMA modelling: model identification, parameter estimation, and model diagnostic checking. After stationarity of the series has been verified through Augmented Dickey-Fuller (ADF) test and/or Kwiatkowski–Phillips–Schmidt–Shin (KPSS) test, the autoregressive (AR) and moving average (MA) components of the ARIMA model will be identified using autocorrelation function (ACF) and partial autocorrelation function (PACF) plots. By changing various parameters for AR and MA components, different models can be constructed and tested. In the situation of two or more models fit with the available data in an acceptable framework, then model selection criteria can be used to select the best model. Some of the widely used model selection criteria are Akaike Information Criteria (AIC), Bayesian Information Criteria (BIC), and Corrected Akaike Information Criteria (AICc). The model which has the minimum AIC, BIC, and AICc measures can be selected as the best ARIMA model. In model diagnostic checking, residuals should follow a white noise which is drawn from a zero mean and a constant variance. Autoregressive Conditional Heteroscedasticity-Lagrange Multiplier (Arch LM) test with the null hypothesis of residuals of the ARIMA model are homoscedastic can be used to validate the constant variance assumption. If the assumptions do not hold, then another model needs to be investigated; otherwise, the model can be used to make predictions.

#### 2.2.3. Mean Absolute Percentage Error (MAPE)

The MAPE value is widely applied in testing the accuracy of the forecasts generated by models. If the MAPE value is less than 10%, then the corresponding model has higher accuracy in forecasting [12].

(3) $$\begin{array}{c} \text{MAPE}=\frac{100}{n}\sum \left|\frac{y_{i}{\hat{y}}_{l}}{y_{i}}\right|, \end{array}$$

where *n* is the number of observations and $y_{i},{\hat{y}}_{l}$ are the actual and predicted values, respectively.

#### 2.2.4. Paired *t*-test

The paired sample *t*-test (dependent sample *t*-test) is usually applied to test whether the mean difference of two related groups is zero or not. Therefore, the null hypothesis is the mean difference between the related samples is zero. The upper tailed and lower tailed alternative hypothesis can be set as the mean difference is greater than zero and less than zero. The test assumes that the dependent variable is normally distributed. The Shapiro-Wilk normality test is useful in assessing the normality assumption, where the null hypothesis is the data are normally distributed. The paired samples Wilcoxon test can be used as an alternative to the paired sample *t*-test if the data are not normally distributed.

The test statistic of the paired *t*-test is given as:

(4) $$\begin{array}{c} \frac{\bar{d}-\mu_{d}}{\bar{s}/\sqrt{n}}, \end{array}$$

where $\bar{d}$ is the sample mean difference, *μ*_*d*_ is the population mean difference, $\bar{s}$ is the standard deviation of the differences, and *n* is the sample size. Under the null hypothesis, this statistic follows a *t*-distribution with *n* − 1 degrees of freedom.

## 3. Results and Interpretations

**Figure 1:**
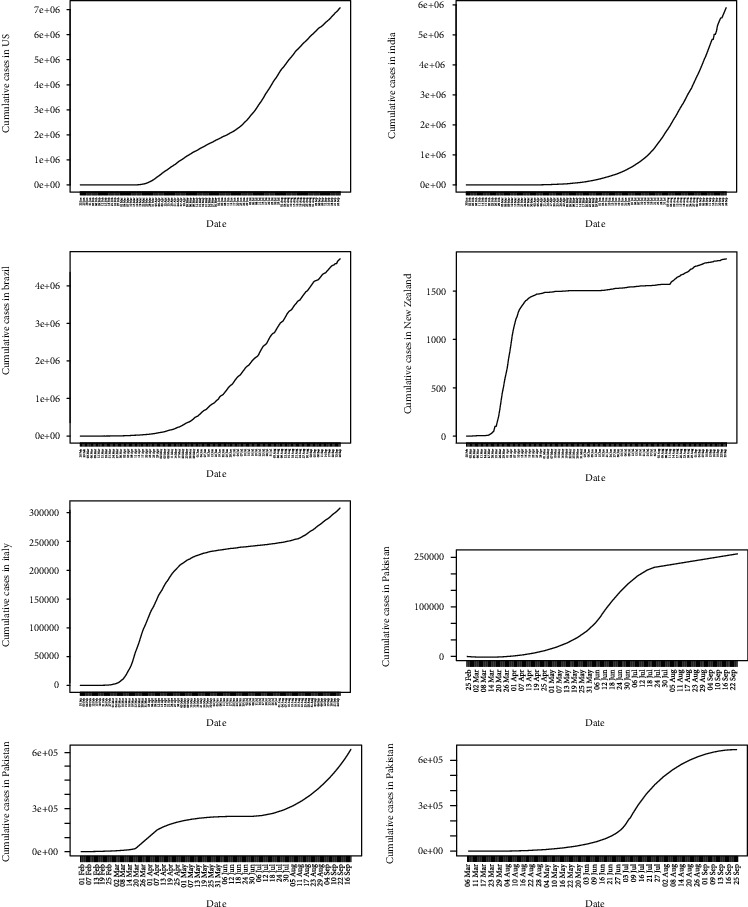
Cumulative COVID-19 cases.

The daily accumulated COVID-19 cases in the eight countries are shown in Figure 1. COVID-19 cases in India and Spain seem to be rapidly increasing. The USA and Brazil are still in the growing phase of the epidemic, and therefore, further transmissions are possible. In New Zealand, the epidemic seems to be saturated, and in Italy, it is increasing further during their second wave. In Pakistan and South Africa, the epidemic is increasing at a lower rate.

**Figure 2:**
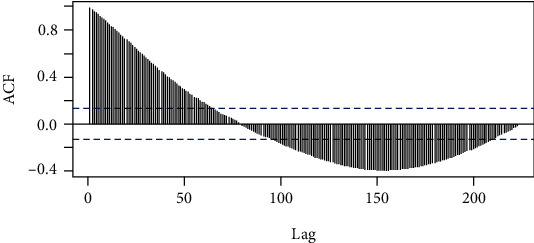
ACF of the original series of the USA.

ARIMA models were fit for data of each country with the aim of predicting future cases. The ACF plot of the data in the USA is shown in Figure 2. Both ADF and KPSS confirmed the nonstationarity of the series at 5% significance level. The differencing method was applied to remove the nonstationarity. Second-order differencing removed the nonstationarity, and stationarity of the resulted series was confirmed by the ADF and KPSS tests. Then, by changing various parameters for MA and AR components of ARIMA, numerous models were constructed and summarized in Table 3.

**Figure 3:**
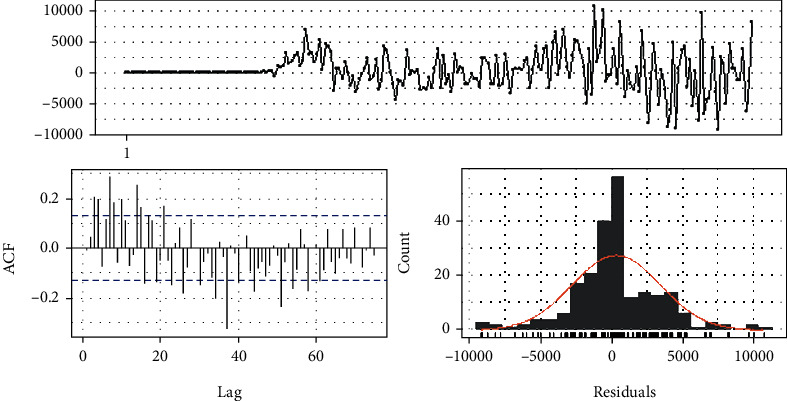
Residual analysis of ARIMA (3,2,3).

The ARIMA (3,2,3) model consists of minimum AIC, BIC, and AICc measures. Therefore, the selected model is tested for model assumptions. Residual analysis of the model is given in Figure 3. Box-Ljung test on residuals as well as on squared residuals was not statistically significant at 5% significance level. The Jarque Bera test revealed the nonnormality of the residuals, and the Arch LM test on residuals confirmed the heteroscedasticity of residuals.

Therefore, the selected model which has the minimum summary measures was not appropriate, and the second and third ARIMA models which have the next lowest AIC, BIC, and AICc values were checked for assumptions. Assumptions were not satisfied by the models, and the summarized results are shown in Table 4.

Three models which have minimum AIC, BIC, and AICc measures do not satisfy the assumptions. There is a presence of heteroscedasticity in the residuals. Hence, simple ARIMA modelling approach may not be adequate, and ARIMA-ARCH modelling may be appropriate in modelling cumulative COVID-19 cases in the USA.

**Figure 4:**
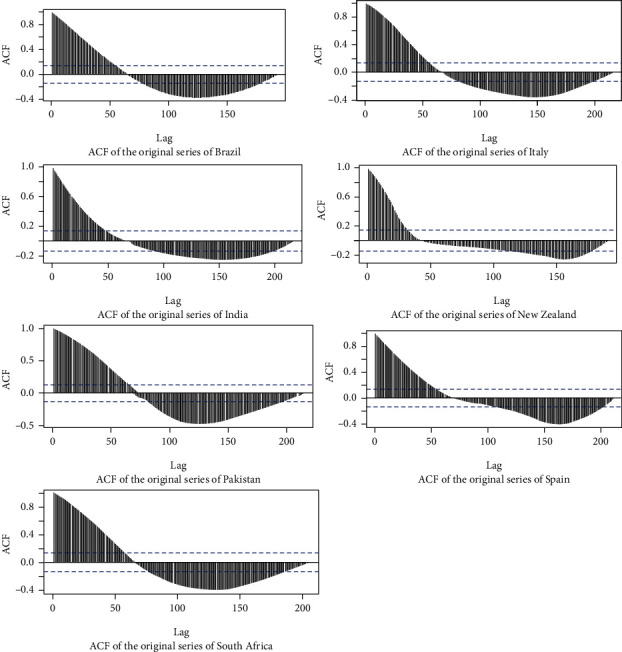
ACF plots of the original series of countries.

The same approach was implemented in the training data series of other countries. The ACF plots of the series are given in Figure 4.

Second-order differencing removed the nonstationarity in data of all the selected countries, and stationarity of the resulted series were confirmed by the ADF and KPSS tests. Then, by changing various parameters for MA and AR components of ARIMA, numerous models were constructed for each country and summarized in Supplementary Materials as Tables 1–7.

**Figure 5:**
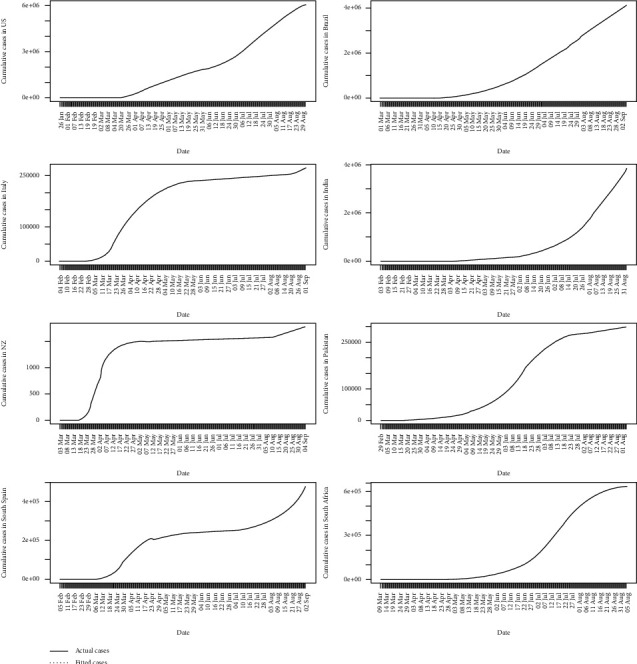
Actual and fitted values—training set.

**Figure 6:**
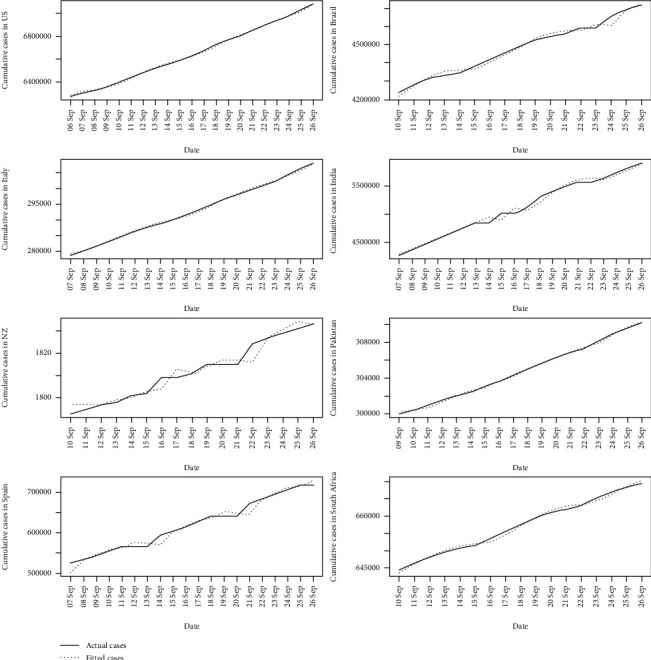
Validation plots in each of the countries.

The best model for each country was selected which has minimum AIC, BIC, and AICc measures. The residual analysis of the best model in each country was included in the supplementary materials from Figures 1–7. All of the assumptions of residuals were not satisfied by the best models, and then, two other candidate models were further tested for each country. The summary of the residual analysis is given in Table 5.

Three models for each country which has minimum AIC, BIC, and AICc measures do not satisfy all the assumptions. There is a presence of heteroscedasticity in the residuals of all models excluding ARIMA models for India. Hence, ARIMA-ARCH modelling may be appropriate in modelling cumulative COVID-19 cases in Italy, New Zealand, Brazil, Pakistan, Spain, and South Africa. For ARIMA, models in India do not satisfy assumptions of no autocorrelations and normality. Therefore, fitted models are not appropriate in generating forecasts for series in India. Hybrid models may be able to capture the remaining patterns exist in the residuals.

The Alpha-Sutte modelling approach was utilized with the aim of modelling and predicting COVID-19 cases. The fitted Alpha-Sutte models and the actual COVID-19 case values for the eight countries in the training data set are shown in Figure 5. It is apparent that the fitted values are following the actual data values in the selected countries.

The last 10% of the data were used to validate the Alpha-Sutte Indicator approach. Therefore, in the validation set, the data starts from September 2, 2020, for the USA; September 6, 2020, for Brazil, South Africa, and New Zealand; September 5, 2020, for Pakistan; and September 3, 2020, for India, Spain, and Italy. The last date of data recording for all the selected countries was September 26, 2020. The validation plots are given in Figure 6.

The calculated MAPE values in the validation set for each country are given in Table 6.

As the percentage errors are less than 10% [12], the Alpha-Sutte Indicator method has higher accuracy in forecasting the cumulative COVID-19 cases in the USA, Brazil, Italy, India, Spain, South Africa, Pakistan, and New Zealand. Furthermore, the Shapiro-Wilk normality test confirmed the normality of the differences of actual and predicted values in all the selected countries except differences in Spain and New Zealand (Table 7). The paired *t*-test was applied for actual and fitted values in the validation data series in each of the countries excluding New Zealand and Spain. The results of the paired *t*-test are summarized in Table 8.

The nonparametric paired samples Wilcoxon test was applied to check whether there is a median difference of actual and predicted values in the data of New Zealand and Spain. The *p* values of the Wilcoxon test are 0.349 and 0.229 for the data of Spain and New Zealand, respectively. Therefore, there is no median difference in the actual and predicted values in the data series in Spain and New Zealand. The mean difference (or the median difference) of actual values and predicted values was not statistically significant at the 5% level of significance, emphasizing that the Alpha-Sutte approach performed well in modelling COVID-19 cases in the selected countries. Further, actual and predicted values in the validation data set of all the countries were highly correlated to each other. The forecasted value generated for the next data point (September 27, 2020) was summarized in Table 9.

## 4. Conclusions and Recommendations

This study successfully models the cumulative COVID-19 case counts exhibited in the USA, Brazil, Italy, India, Pakistan, South Africa, Spain, and New Zealand with the aim of modelling and predicting COVID-19 cases. Selection of the countries was based on the different growing phases of the COVID-19 epidemic as well as various social and geographical characteristics present in countries. Alpha-Sutte Indicator approach and ARIMA modelling approach were selected for the modelling purpose.

All the best-fitted ARIMA models which have minimum AIC, BIC, and AICc measures do not satisfy all the assumptions of the residuals. The residuals of the best-fitted models for most of the countries exhibited heteroscedasticity, and ARIMA-ARCH modelling may be appropriate. Further, hybrid modelling approaches by coupling ARIMA with another modelling technique may be appropriate in capturing the remaining patterns which exist in the residuals. Therefore, a simple ARIMA modelling approach is not adequate in modelling and predicting the cumulative COVID-19 cases present in the selected eight countries for the period considered in the study. The analysis demonstrated the powerfulness of the Alpha-Sutte modelling approach over the ARIMA method in modelling cumulative COVID-19 cases in the selected eight countries.

The Alpha-Sutte modelling approach requires only few observations in modelling, and no assumptions are existing to validate the predictions that were generated. The method is simple and easy to understand. No advanced mathematical knowledge or subject-related background are required to implement the method. Further, users can implement the method without using any specific statistical, mathematical, or programming languages or packages/software. These features enhance the applicability of the modelling strategy as many conventional univariate time series modelling approaches such as ARIMA modelling require more than 50 observations in modelling, and certain assumptions should be satisfied to validate the predictions. In addition, statistical, mathematical, or programming software packages are needed to succeed in the application of ARIMA and other univariate time series modelling approaches. The capability of the Alpha-Sutte method in modelling cumulative COVID-19 cases over ARIMA modelling was illustrated in the study. The analysis revealed that the Alpha-Sutte Indicator method has a higher accuracy in forecasting the cumulative COVID-19 cases in the USA, Brazil, Italy, India, New Zealand, Pakistan, Spain, and South Africa by reporting 0.11%, 0.33%, 0.08%, 0.72%, 0.12%, 0.03%, 1.28%, and 0.08% of the mean absolute percentage errors (MAPE), respectively. Furthermore, the paired *t*-test confirmed that there are no differences between real COVID-19 incidence and forecasted incidence through Alpha-Sutte approach at the 5% level of significance in each of the selected countries. Thus, predictions were generated using the Alpha-Sutte approach for each country. It can be concluded from the analysis that the Alpha-Sutte approach outperformed the ARIMA approach in modelling cumulative COVID-19 cases presented in the eight countries. Although the Alpha-Sutte method is simple, the effectiveness and efficiency of the method is high and unchallengeable compared to the ARIMA technique in terms of modelling cumulative COVID-19 cases. Therefore, the Alpha-Sutte method can be recommended for short-term forecasting of cumulative COVID-19 incidences irrespective of the growing stage of the epidemic and socioeconomic and geographical characteristics of countries.

The predictions of COVID-19 cases can provide tremendous advantages for countries. Some of them include, as to implement and enhance controlling actions, to handle the process cost-effectively, to discover more opportunities and possibilities to manage the epidemic, proper management of health-related resources, identify mistakes and drawbacks to eventually minimize the hazards, etc. Therefore, the prediction of the Alpha-Sutte approach will direct endless opportunities for countries. Particularly, the Alpha-Sutte method can be used to answer the question “what will be the COVID-19 cases tomorrow or the next time period?”. The COVID-19 cases are rapidly changing, and the knowledge of what will happen in the next time period is important in handing all the resources associated with COVID-19. Quick decisions are needed to mitigate the impact of the epidemic, and the Alpha-Sutte method is supportive in the objective. The method can be implemented in the local or regional level as well. The method adjusts its parameters to generate the prediction at the next time period when sudden increases or decreases come across.

Furthermore, predictions are always associated with uncontrollable and uncountable uncertainties. Sudden spikes in the number of daily reporting may indicate due to imported cases. Unexpected clusters of COVID-19 cases may come across due to holes existing in the screening processes. Such uncertainties are limiting the productivity and throughput of a modelling process.

## Figures and Tables

**Table 1:** Some socioeconomic and geographical characteristics of the selected countries.

Country	Population	Number of 65+ aged population (in millions)	Land area (km^2^)	Share of world GDP (%)(2017)	Expenditure on education (% of GDP)	Human development index	Continent
Spain	46,761,579	8.99	498,800	1.62	4.2 (2016)	0.891	Europe
Italy	60,428,372	13.76	294,140	2.4	3.8 (2016)	0.88	Europe
New Zealand	5,002,100	0.76	263,310	0.25	6.4 (2016)	0.917	Oceania
USA	331,727,464	52.76	9,147,420	24.08	5.0 (2014)	0.924	North America
India	1,385,049,027	84.9	2,973,190	3.28	3.1 (2019)	0.64	Asia
Pakistan	222,469,681	9.31	770,880	0.38	2.9 (2017)	0.562	Asia
Brazil	213,122,892	17.79	8,358,140	2.54	6.2 (2015)	0.759	South America
South Africa	59,585,858	3.51	1,213,090	0.43	6.2 (2018)	0.699	Africa

**Table 2:** Starting date of data recording of the countries.

Country	Starting date of the year 2020	Closing date of the data in the training data set
Italy	January 31	September 2
New Zealand	February 28	September 5
USA	January 22	September 1
Brazil	January 26	September 5
Pakistan	February 25	September 4
Spain	February 1	September 2
South Africa	March 5	September 5
India	January 30	September 2

**Table 3:** Summary measures of candidate ARIMA models—USA series.

Model	BIC	AIC	AICc
ARIMA (2,2,2)	4,217	4,200	4,200.5
ARIMA (0,2,0)	4,298	4,294.6	4,294.6
ARIMA (1,2,0)	4,303.9	4,297	4,297
ARIMA (0,2,1)	4,302.9	4,296	4,296
ARIMA (1,2,2)	4,303.3	4,289.7	4,289.8
ARIMA (2,2,1)	4,301	4,287.6	4,287.8
ARIMA (3,2,2)	Infinity	Infinity	Infinity
ARIMA (2,2,3)	4,303	4,282.6	4,282.9
ARIMA (1,2,1)	4,300	4,290	4,290
ARIMA (1,2,3)	4,296.6	4,279.6	4,279.8
ARIMA (3,2,1)	4,277.7	4,260.7	4,260.9
ARIMA (3,2,3)	4,207.7	4,183.9	4,184
ARIMA (4,2,3)	Infinity	Infinity	Infinity
ARIMA (3,2,4)	4,212.9	4,185.7	4,186
ARIMA (2,2,4)	Infinity	Infinity	Infinity
ARIMA (4,2,2)	Infinity	Infinity	Infinity
ARIMA (4,2,4)	4,218	4,187.6	4,188

**Table 4:** Results of residual analysis—USA series.

Model	Box-Ljung test on residuals(*p* value)	Box-Ljung test on squared residuals(*p* value)	Jarque Bera test(*p* value)	Arch LM test(*p* value)
ARIMA (3,2,3)	2.2e-16	0.01489	7.598e-07	≤0.001
ARIMA (3,2,4)	2.2e-16	0.02482	4.53e-08	≤0.001
ARIMA (4,2,4)	2.2e-16	0.04135	3.205e-08	≤0.001

**Table 5:** Results of residual analysis.

Country	Model	Box-Ljung test on residuals(*p* value)	Box-Ljung test on squared residuals(*p* value)	Jarque Bera test(*p* value)	Arch LM test(*p* value)
Brazil	ARIMA (3,2,2)	2.2e-16	0.0072	2.2e-16	5.4826e-05
	ARIMA (2,2,2)	2.2e-16	0.0017	2.2e-16	8.2622e-06
	ARIMA (4,2,1)	2.2e-16	0.0008	2.2e-16	3.0266e-07

Italy	ARIMA (2,2,2)	2.39e-11	2.98e-07	2.2e-16	≤0.001
	ARIMA (2,2,3)	9.36e-12	1.59e-07	2.2e-16	≤0.001
	ARIMA (3,2,3)	1.88e-12	2.09e-07	2.2e-16	≤0.001

India	ARIMA (0,2,2)	2.2e-16	0.3965	2.2e-16	0.3481
	ARIMA (1,2,0)	2.2e-16	0.5325	2.2e-16	0.2552
	ARIMA (0,2,3)	2.2e-16	0.3970	2.2e-16	0.3469

New Zealand	ARIMA (0,2,2)	0.076	7.76e-11	2.2e-16	≤0.001
	ARIMA (0,2,3)	0.055	4.23e-11	2.2e-16	≤0.001
	ARIMA (1,2,2)	0.055	5.37e-11	2.2e-16	≤0.001

Pakistan	ARIMA (0,2,4)	0.062	0.0320	2.2e-16	2e-08
	ARIMA (0,2,5)	0.059	0.019	2.2e-16	3.28e-09
	ARIMA (1,2,3)	0.044	0.028	2.2e-16	3.66e-08

Spain	ARIMA (2,2,4)	2.2e-16	4.78e-09	2.2e-16	≤0.001
	ARIMA (2,2,5)	2.2e-16	6.21e-09	2.2e-16	≤0.001
	ARIMA (3,2,4)	2.2e-16	7.38e-09	2.2e-16	≤0.001

South Africa	ARIMA (2,2,2)	7.95e-12	0.0007	2.2e-16	≤0.001
	ARIMA (3,2,2)	2.2e-16	0.0005	2.2e-16	≤0.001
	ARIMA (3,2,1)	2.2e-16	0.057	2.2e-16	1.11e-16

**Table 6:** MAPE values in the validation set—Alpha-Sutte method.

Country	MAPE (%)
Italy	0.08
New Zealand	0.12
Pakistan	0.03
USA	0.11
India	0.72
Brazil	0.33
South Africa	0.08
Spain	1.28

**Table 7:** The results of the Shapiro-Wilk normality test.

Country	*p* value
USA	0.906
Brazil	0.224
Italy	0.604
India	0.213
Pakistan	0.162
South Africa	0.492
Spain	0.001
New Zealand	0.009

**Table 8:** The results of the paired *t*-test.

Country	Sample mean difference	95% confidence interval	*t* value	*p* value
USA	187	(-3,850, 4,224)	0.097	0.92
Brazil	1,232	(-8,608, 11,073)	0.265	0.79
Italy	32.85	(-94, 160)	0.542	0.59
India	-1386	(-26,656, 23,883)	-0.115	0.91
Pakistan	21.61	(-42, 85)	0.712	0.49
South Africa	-26.52	(-357, 303)	-0.17	0.87

**Table 9:** Forecasted value for each of the selected country.

Country	Forecasted value
USA	7,126,437
Brazil	4,760,423
Italy	309,965
India	5,990,546
Pakistan	310,962
South Africa	670,936
Spain	724,185
New Zealand	1,835

## Data Availability

The data used to support the findings of this study are available in HUMANITARIAN DATA EXCHANGE at https://data.humdata.org/dataset/novel-coronavirus-2019-ncov-cases?force_layout=desktop.

## Supplementary Materials

Supplementary Materials The supplementary file consists of Table 1 to Table 7 which consist of candidate ARIMA models with corresponding summary measures (AIC, BIC, and AICc) fitted for Brazil, Italy, India, New Zealand, Pakistan, Spain, and South Africa, respectively. Further, Figure 1 to Figure 7 correspond to the plots of residual analysis of the best-fitted ARIMA model for each of the eight countries.
