# Supplementary material for: Forecasting COVID-19 Cases Using Alpha-Sutte Indicator: A Comparison with Autoregressive Integrated Moving Average (ARIMA) Method
Source: Biomed Res Int. 2020 Dec 3;2020:8850199. doi: 10.1155/2020/8850199 (PMC7733325; doi:10.1155/2020/8850199)

Table 1: Summary Measures of Candidate ARIMA Models – Brazil Series

Model	BIC	AIC	AICc
ARIMA(2,2,2)	3927	3911	3911
ARIMA(0,2,0)	4022	4019	4019
ARIMA(1,2,0)	4028	4021	4021
ARIMA(0,2,1)	4026	4019	4019
ARIMA(1,2,2)	3975	3962	3962
ARIMA(2,2,1)	3964	3951	3951
ARIMA(3,2,2)	3926	3906	3907
ARIMA(3,2,1)	3964	3947	3948
ARIMA(4,2,2)	Infinity	Infinity	Infinity
ARIMA(3,2,3)	Infinity	Infinity	Infinity
ARIMA(2,2,3)	3971	3952	3952
ARIMA(4,2,1)	3939	3919	3920
ARIMA(4,2,3)	Infinity	Infinity	Infinity

Table 2: Summary Measures of Candidate ARIMA Models – Italy Series

Model	BIC	AIC	AICc
ARIMA(2,2,2)	3056	3039	3039
ARIMA(0,2,0)	3081	3078	3078
ARIMA(1,2,0)	3087	3080	3080
ARIMA(0,2,1)	3086	3079	3079
ARIMA(1,2,2)	3095.3	3081.8	3082
ARIMA(2,2,1)	3095.7	3082.3	3082.5
ARIMA(3,2,2)	3064	3043	3043.8
ARIMA(2,2,3)	3061	3040	3041.1

ARIMA(1,1,2)	3091	3081	3081
ARIMA(1,2,3)	3099.9	3083	3083
ARIMA(3,2,1)	3099.4	3082	3082
ARIMA(3,2,3)	3067	3043	3043.8

Table 3: Summary Measures of Candidate ARIMA Models – India Series

Model	BIC	AIC	AICc
ARIMA(2,2,2)	Infinity	Infinity	Infinity
ARIMA(0,2,0)	4027	4023	4023
ARIMA(1,2,0)	4026	4019	4019
ARIMA(0,2,1)	4020	4014	4013
ARIMA(1,2,1)	4021	4010.9	4011
ARIMA(0,2,2)	4018	4008	4008
ARIMA(1,2,2)	4025	4011	4011.3
ARIMA(0,2,3)	4024	4010.1	4010
ARIMA(1,2,3)	4028	4010.9	4011.1

Table 4: Summary Measures of Candidate ARIMA Models – NZ Series

Model	BIC	AIC	AICc
ARIMA(2,2,2)	1295.6	1279	1279.8
ARIMA(0,2,0)	1330	1327	1327
ARIMA(1,2,0)	1288	1282	1282
ARIMA(0,2,1)	1300	1294	1294
ARIMA(0,2,2)	1292	1274	1274
ARIMA(1,2,1)	1293	1283.5	1284

ARIMA(0,2,3)	1292.8	1275.9	1276
ARIMA(1,2,2)	1293	1277	1277

Table 5: Summary Measures of Candidate ARIMA Models – Pakistan Series

Model	BIC	AIC	AICc
ARIMA(2,2,2)	3489	3472.7	3473
ARIMA(0,2,0)	3608	3605	3605
ARIMA(1,2,0)	3578	3571	3571
ARIMA(0,2,1)	3507	3501	3501
ARIMA(1,2,2)	3508	3495	3496
ARIMA(2,2,1)	3488	3475	3475
ARIMA(0,2,4)	3496	3461.6	3461.8
ARIMA(0,2,5)	3483	3462.9	3463.3
ARIMA(1,2,3)	3488	3463.4	3463.7

Table 6: Summary Measures of Candidate ARIMA Models – Spain Series

Model	BIC	AIC	AICc
ARIMA(2,2,2)	4537	4520	4520
ARIMA(0,2,0)	4720	4717	4717
ARIMA(1,2,0)	4677	4670	4670
ARIMA(0,2,1)	4569	4562	4562
ARIMA(1,2,2)	4542	4528	4528
ARIMA(2,2,1)	4551	4537	4538
ARIMA(3,2,2)	4562	4541	4541
ARIMA(2,2,3)	Infinity	Infinity	Infinity
ARIMA(2,2,5)	4505	4477	4477
ARIMA(3,2,4)	4506	4478	4478
ARIMA(2,2,4)	4499	4475	4475
ARIMA(0,2,4)	4535	4518	4518

ARIMA(0,2,3)	4530	4516	4516

Table 7: Summary Measures of Candidate ARIMA Models – South Africa Series

Model	BIC	AIC	AICc
ARIMA(2,2,2)	3325	3309	3309
ARIMA(0,2,0)	3386	3383	3383.3
ARIMA(1,2,0)	3392	3385	3386
ARIMA(0,2,1)	3391	3384	3384.8
ARIMA(1,2,2)	3384	3371	3371.7
ARIMA(2,2,1)	3381	3368	3368
ARIMA(3,2,2)	3330	3310	3310
ARIMA(3,2,1)	3381	3364	3365
ARIMA(3,2,3)	Infinity	Infinity	Infinity
ARIMA(1,2,3)	3388	3372	3372.6
ARIMA(1,2,1)	3385	3375	3376
ARIMA(3,2,2)	Infinity	Infinity	Infinity

Figure 1: Residual Analysis of ARIMA (3,2,2) - Brazil

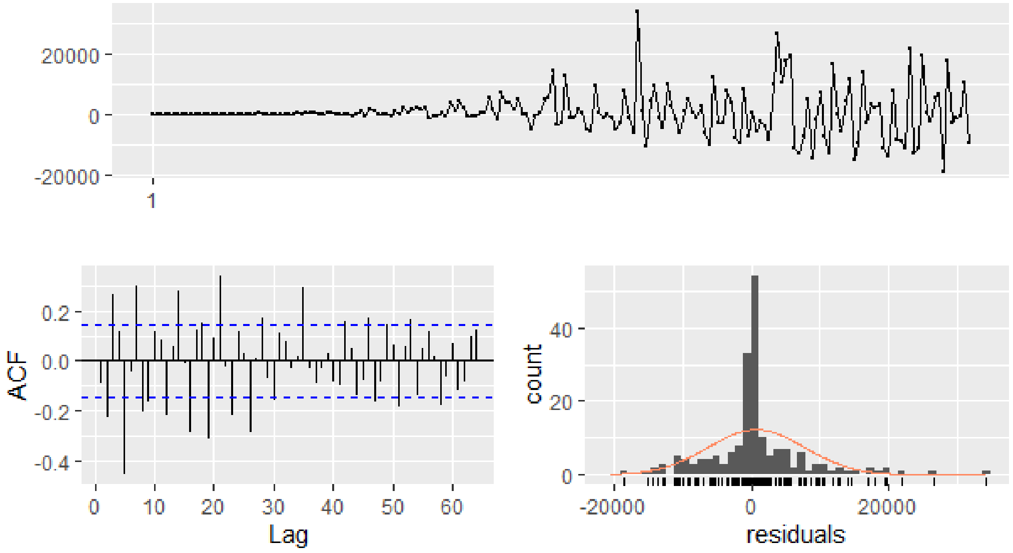

Figure 2: Residual Analysis of ARIMA (2,2,2) - Italy

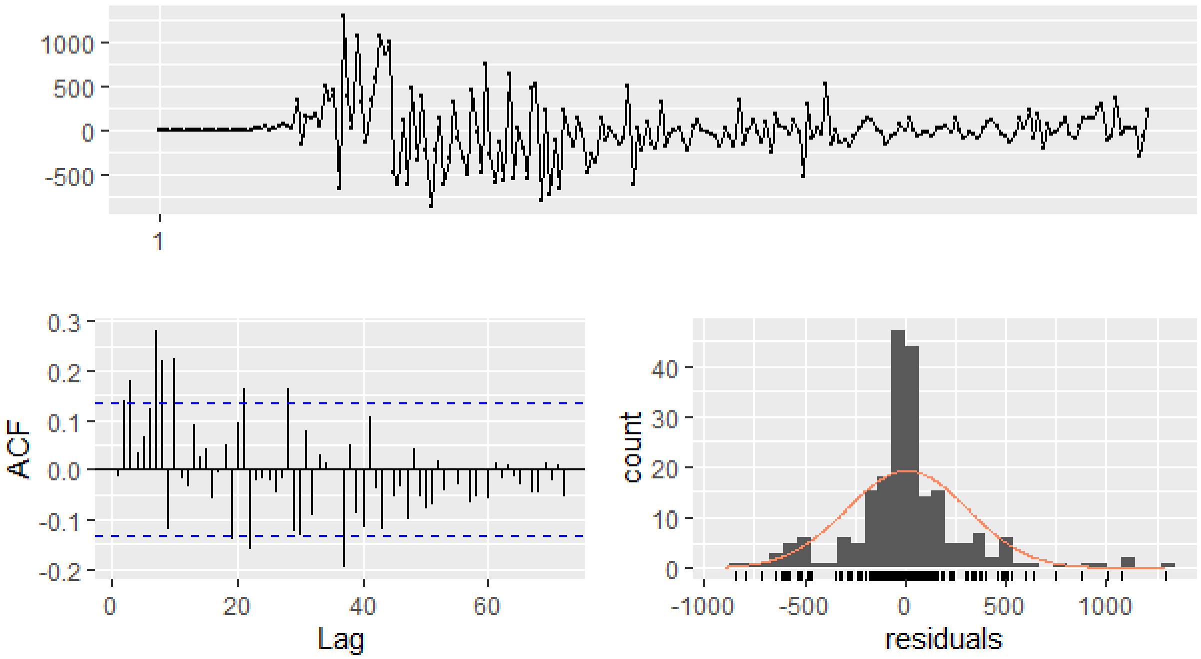

Figure 3: Residual Analysis of ARIMA (0,2,2) - India

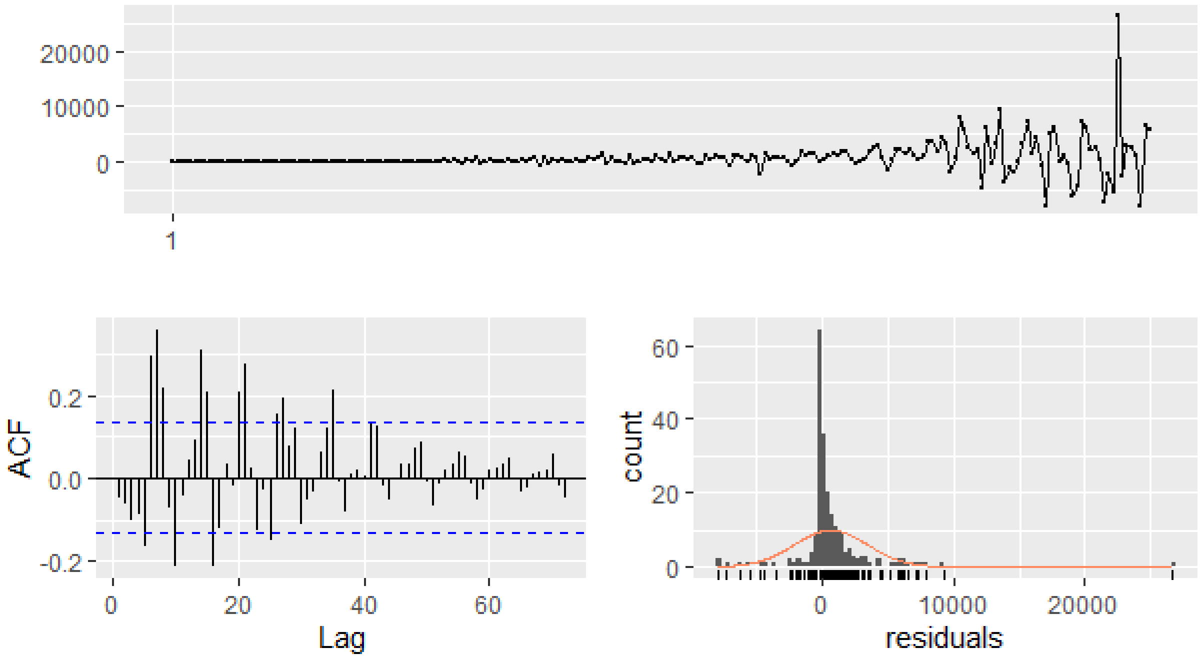

Figure 4: Residual Analysis of ARIMA (0,2,2) – New Zealand

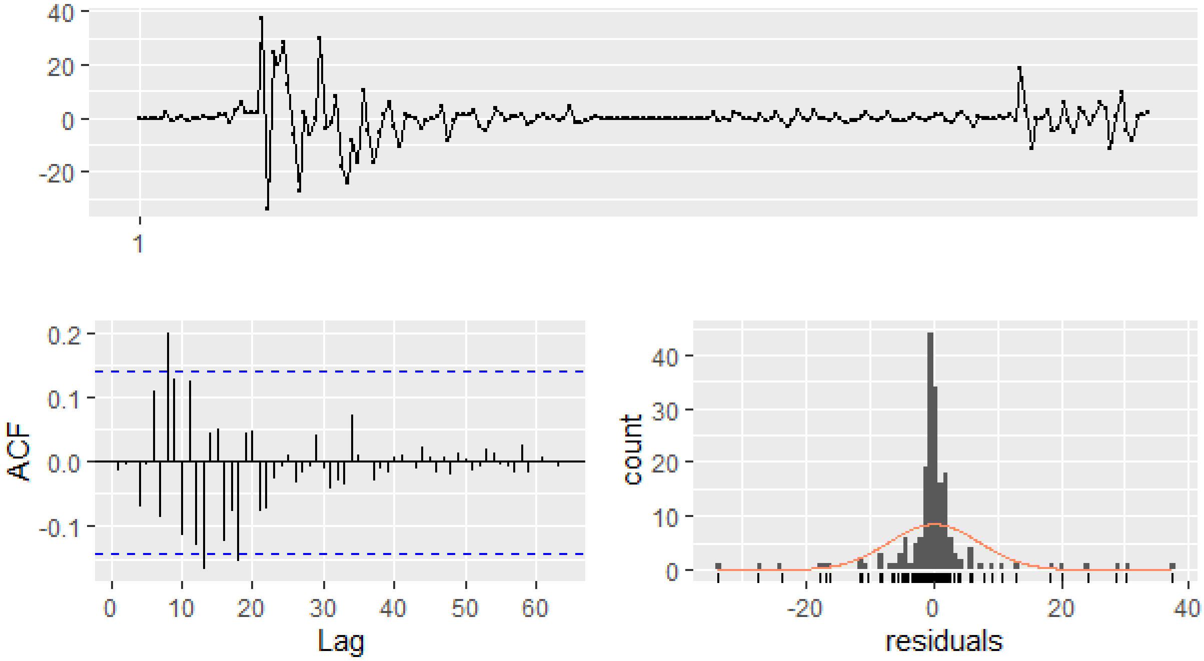

Figure 5: Residual Analysis of ARIMA (0,2,4) – Pakistan

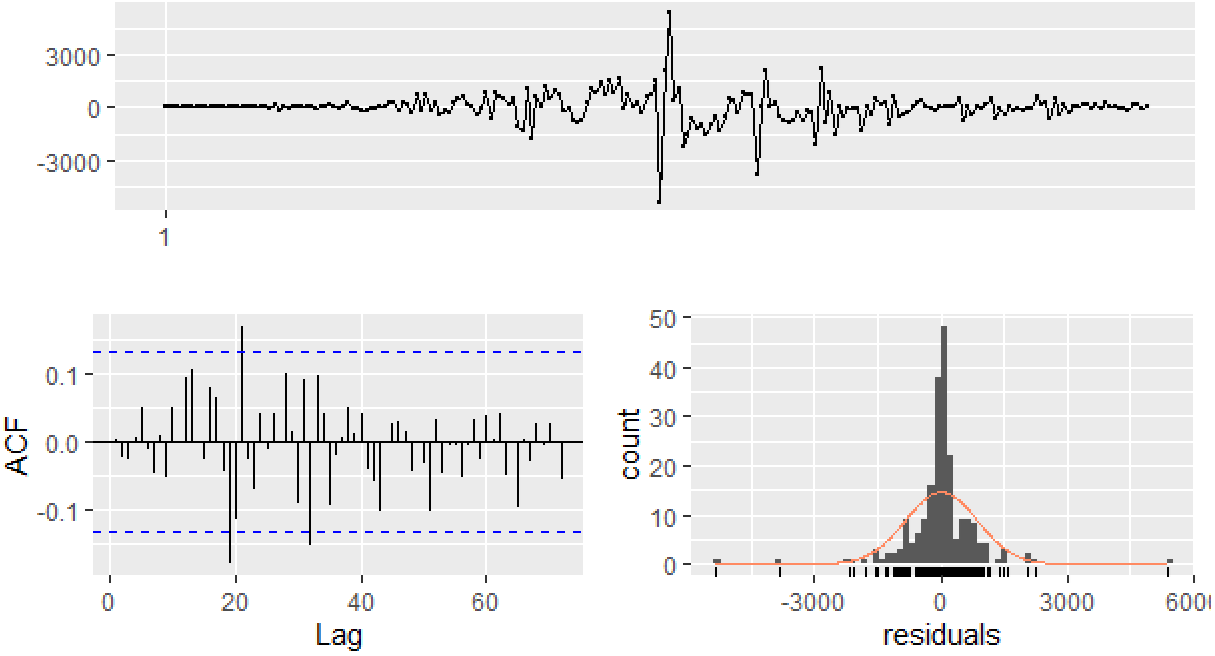

Figure 6: Residual Analysis of ARIMA (2,2,4) – Spain

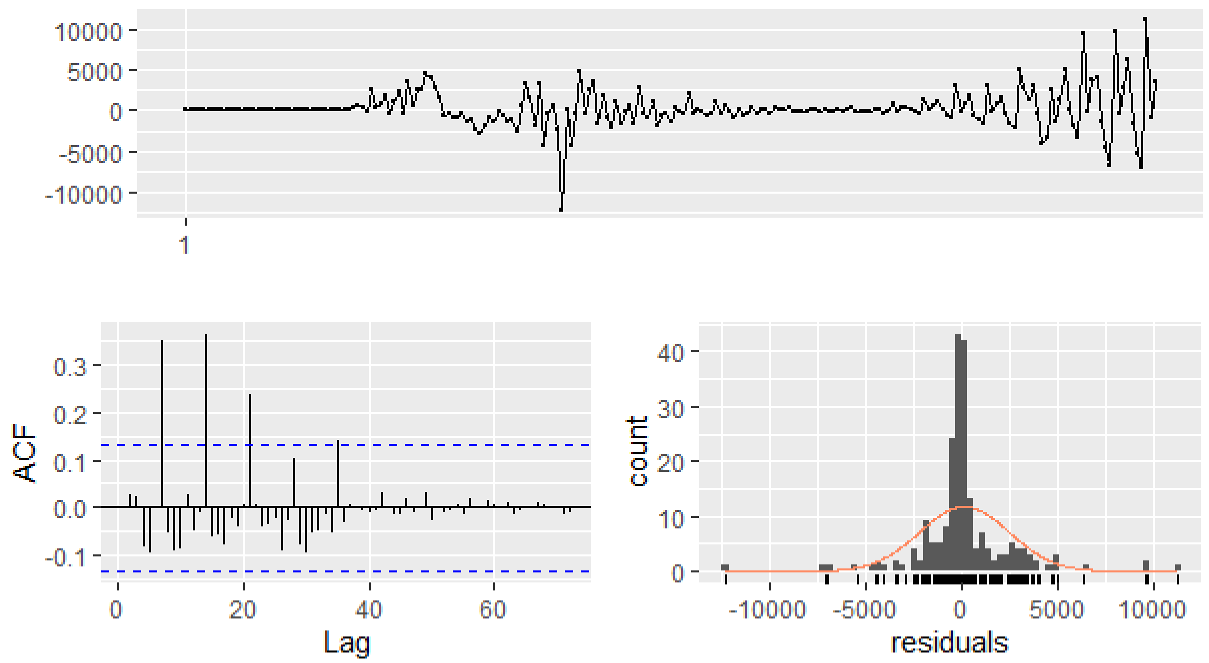

Figure 7: Residual Analysis of ARIMA (2,2,2) – South Africa

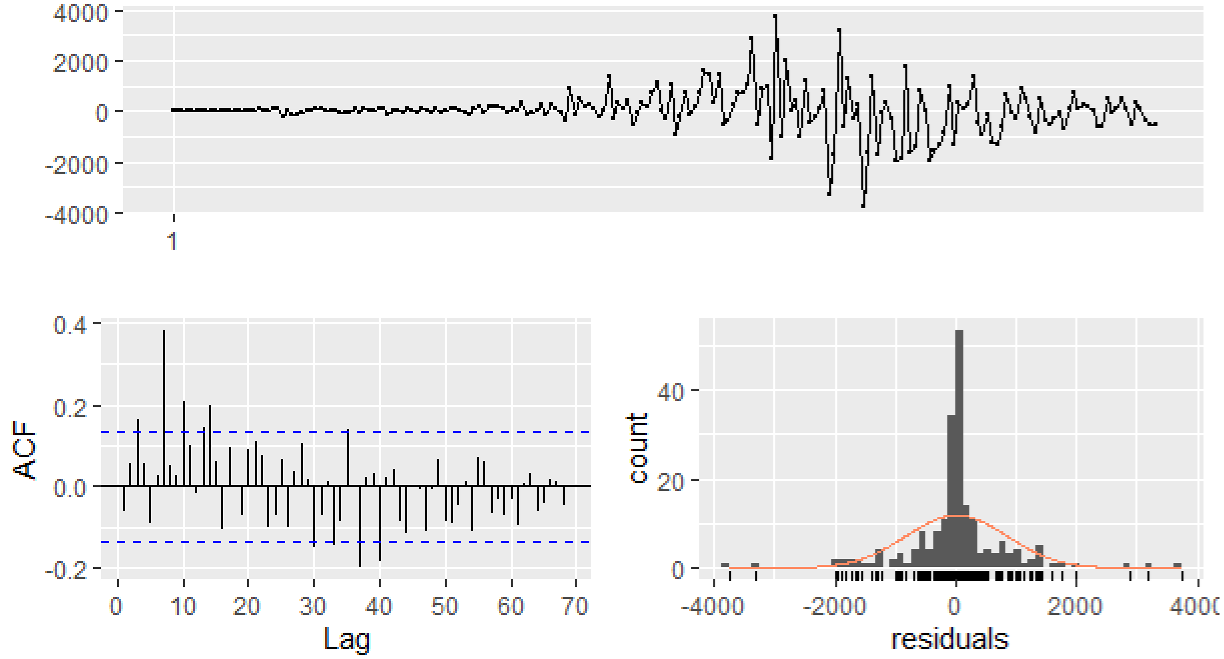

Supplement: Supplementary Materials — The supplementary file consists of Table 1 to Table 7 which consist of candidate ARIMA models with corresponding summary measures (AIC, BIC, and AICc) fitted for Brazil, Italy, India, New Zealand, Pakistan, Spain, and South Africa, respectively. Further, Figure 1 to Figure 7 correspond to the plots of residual analysis of the best-fitted ARIMA model for each of the eight countries. [file 8850199.f1.pdf]
